# Biological activity of *Juglans regia* leaf extract and *in silico* evaluation of its phytochemicals against *Plasmodium berghei* target proteins

**DOI:** 10.2478/helm-2026-0009

**Published:** 2026-06-15

**Authors:** H. M. ALHARBI, M. DKHIL, S. SANTOURLIDIS, M. ALBESHR, E. M. AL-SHAEBI, R. ABDEL-GABER

**Affiliations:** 1 Department of Biology, College of Science, Princess Nourah bint Abdulrahman University, P.O. Box 84428, Riyadh 11671, Saudi Arabia; 2 Department of Zoology and Entomology, Faculty of Science, Capital University (formerly Helwan University), Cairo, Egypt; 3 Epigenetics Core Laboratory, Institute of Transplantation Diagnostics and Cell Therapeutics, Medical Faculty, Heinrich-Heine University Düsseldorf, Düsseldorf, Germany; 4 Department of Zoology, College of Science, King Saud University, P.O. 2455, Riyadh 11451, Saudi Arabia

**Keywords:** *Juglans regia*, anthelmintic, *Eisenia fetida*, molecular docking, *Plasmodium berghei*, phytochemicals

## Abstract

Parasitic infections remain a major global health challenge, with helminthiasis causing significant morbidity and economic losses. Limitations of synthetic anthelmintics, such as resistance and toxicity, have encouraged the search for natural plant-based alternatives. *Juglans regia* (walnut) leaves are rich in bioactive phytochemicals with potential antiparasitic effects. This study aimed to evaluate the anthelmintic efficacy of the methanolic leaf extract of *J. regia* (JRLE) against *Eisenia fetida* and to assess, through molecular docking, the potential interactions of its phytochemicals with selected *Plasmodium berghei* proteins. Fresh *J. regia* leaves were extracted with 70 % methanol using a maceration technique for 48 h at room temperature to obtain the crude extract. Adult *E. fetida* worms were treated with JRLE at concentrations of 50, 100, and 200 mg/ml, using mebendazole (10 mg/ml) as a reference and distilled water as a control. Paralysis and death times were recorded, and histological examinations were conducted to assess structural alterations in the worm cuticle. Phytochemicals identified in JRLE were docked against *P berghei* target proteins (Profilin, L-lactate dehydrogenase, Cytochrome c oxidase, Cytochrome b, and Pyridoxal 5’-phosphate synthase subunit) using Schrodinger Glide XP. JRLE exhibited significant dose-dependent anthelmintic activity, with the 200 mg/ml concentration inducing paralysis and death faster than mebendazole. Histological findings revealed cuticular erosion, epidermal vacuolization, and partial detachment of the cuticle from underlying tissues in treated worms. Molecular docking analysis revealed favorable binding interactions of compounds such as γ-sitosterol, stigmasterol, 3-O-methyl-D-glucose, and 4H-pyran-4-one derivatives with *P. berghei* target proteins, with docking scores ranging from approximately -4.494 to -9.799 kcal/mol, indicating stable ligand-protein complexes and suggesting potential antiplasmodial activity based on the *in silico* analysis. These findings demonstrate that JRLE exhibits promising anthelmintic activity and shows *in silico* evidence of potential antiplasmodial effects; however, further *in vitro* and in vivo investigations are necessary to validate the efficacy of its phytochemicals against *Plasmodium* species.

## Introduction

Parasitic infections continue to pose a major global health challenge, affecting both humans and animals, particularly in tropical and subtropical regions (Ndjonka *et al*., 2023; Alhaiqi *et al*., 2024). Among these, helminthiasis caused by parasitic worms is one of the most prevalent parasitic diseases, contributing to malnutrition, impaired growth, and substantial economic losses in livestock production (Halton, 2004). The extensive and often indiscriminate use of synthetic anthelmintics, such as benzimidazoles (e.g., albendazole), has led to the emergence of drug-resistant parasite strains, thereby reducing the efficacy of conventional chemotherapeutics (Chartier *et al*., 2001). Furthermore, issues related to drug toxicity, environmental persistence, and high treatment costs have intensified the search for safe, cost-effective, and eco-friendly alternatives derived from natural sources (Enechi *et al*., 2019; Shi *et al*., 2022).

To overcome these challenges, medicinal plants have long been recognized as valuable sources of bioactive phytochemicals with diverse pharmacological properties, including antioxidant, anti-inflammatory, antimicrobial, and antiparasitic activities (Agrawal *et al*., 2010; Ameen *et al*., 2018; Garcia-Bustos *et al*., 2019; Elouafy *et al*., 2023). Among these, *Juglans regia* L. (walnut tree), belonging to the Juglandaceae family, is widely distributed across Asia, Europe, and the Middle East and is commonly cultivated and available in several Middle Eastern regions, including the area where the plant material used in this study was obtained, highlighting its regional accessibility and ethnobotanical relevance. Traditionally, walnut leaves have been used in folk medicine to treat skin disorders, gastrointestinal disturbances, and microbial infections (Igbayilola *et al*., 2022). Phytochemical analyses have revealed that *J. regia* leaves are rich in phenolic acids, flavonoids, tannins, saponins, and sterols—compounds with demonstrated therapeutic potential against a broad range of pathogens (Ara *et al*., 2023). These bioactive constituents make *J. regia* a promising candidate for the development of plant-based antiparasitic agents.

Beyond helminths, protozoan parasites, such as *Plasmodium* species, also represent significant global health threats. In recent years, the integration of computational approaches—particularly molecular docking—has revolutionized early drug discovery by allowing precise prediction and visualization of molecular interactions between bioactive compounds and biological targets (Agu *et al.,* 2023). This *in silico* method evaluates the binding affinity, orientation, and conformational dynamics of ligands within protein active sites, thereby elucidating possible mechanisms of action and complementing *in vitro* and *in vivo* assays. Molecular docking significantly accelerates the identification of lead compounds by reducing time, cost, and experimental resources (Challapa-Mammani *et al*., 2023; Paul *et al*., 2024; Dong *et al*., 2025). Notably, this computational strategy has become a cornerstone in antiparasitic research, where it has been employed to identify potential inhibitors of essential enzymes in protozoan and helminth parasites. For example, studies on *Plasmodium* species have demonstrated their effectiveness in predicting the interactions of natural and synthetic compounds with key targets such as dihydrofolate reductase, lactate dehydrogenase, and cytochrome bc1 complex (Nwonuma *et al*., 2023; Lobato-Tapia *et al*., 2023).

In this study, the anthelmintic activity of the methanolic leaf extract of *J. regia* (JRLE) was evaluated using *Eisenia fetida* as an *in vitro* model, which is commonly employed as a preliminary screening tool due to its physiological similarities to parasitic helminths. Additionally, the potential molecular interactions of JRLE-derived phytochemicals with selected *Plasmodium berghei* target proteins were explored through molecular docking analysis. By combining experimental and computational approaches, this study aims to elucidate the pharmacological potential of *J. regia* as a natural source of anthelmintic and antiplasmodial agents, thereby contributing to the search for sustainable, safe, and effective plant-based therapies against parasitic diseases.

## Materials and Methods

### Plant collection

Fresh leaves of the walnut tree (*Juglans regia*) were collected from the Al Bahah region, Saudi Arabia (20.0°N, 41.5°E), in March 2025 for use in this study. The plant material was taxonomically verified and authenticated at the Herbarium of the Botany Department, King Saud University, to ensure accurate identification and classification. A voucher specimen was deposited under the number KSU-21595 to support proper documentation and future reference.

### Preparation of *J. regia* leaf extracts (JRLE)

Walnut leaves were air-dried at room temperature to allow gradual moisture evaporation while preserving their phytochemical constituents. The dried material was finely powdered using a Hummer Grinder (Edison Electric, ED-CG1400, China) to ensure homogeneity. Approximately 100 g of the powdered leaves was macerated in 1,000 ml of 70 % methanol with gentle agitation for 24 hours at room temperature under light-protected conditions to preserve the stability of bioactive compounds. The mixture was then filtered through Whatman No. 1 filter paper to separate the solvent extract from the plant residue. The filtrate was concentrated under reduced pressure using a rotary vacuum evaporator (Büchi, Switzerland) at 45 °C to yield the crude methanolic extract. The dried extract was subsequently reconstituted in distilled water at the required weight-to-volume ratio to obtain working concentrations for further experimental use.

### Anthelmintic activity

The anthelmintic potential of *J.* leaf extract (JRLE) was evaluated using adult earthworms (*Eisenia fetida*). Before experimentation, the worms were washed thoroughly with distilled water and acclimatized at ambient temperature for 30 minutes. The species was identified and authenticated by a specialist from the College of Food and Agriculture Sciences, King Saud University. *E. fetida* was selected due to its anatomical and physiological similarities to human intestinal roundworms, making it a suitable model for anthelmintic screening.

Test solutions of the methanolic extract were prepared at concentrations of 50, 100, and 200 mg/ml. Mebendazole (Saudi Pharmaceutical Industries, Riyadh, Saudi Arabia) served as the standard reference drug (10 mg/ml), while distilled water acted as the control. The worms were randomly divided into five groups (n = 5 per group) of comparable size (~5 cm) as follows:

**Group 1:** Control, received distilled water.

**Group 2:** Standard, received mebendazole (10 mg/ml).

**Group 3:** JRLE at 50 mg/ml.

**Group 4:** JRLE at 100 mg/ml.

**Group 5:** JRLE at 200 mg/ml.

Each experiment was performed in triplicate. Each group was placed in a separate Petri dish containing the respective test or control solutions. The worms were observed continuously, and the time to paralysis and death was recorded for each worm. Paralysis was defined as the complete loss of movement, except upon vigorous shaking. At the same time, death was confirmed by the absence of movement even when immersed in warm water (50 °C) and by subsequent discoloration of the body. The procedure followed the method described by Parida *et al*. (2010).

### Histological examination and cuticle thickness

Following the paralysis and death assays, both treated and control worms were immediately processed for histological evaluation following the method of Drury and Wallington (1973). Briefly, the specimens were fixed in 10 % formalin for 24 hours, dehydrated through a graded ethanol series, and embedded in paraffin wax. Tissue sections of 5 μm thickness were obtained using a microtome, stained with hematoxylin and eosin (H…E), and subsequently examined and photographed under an Olympus BX61 microscope (Tokyo, Japan).

#### In silico docking study

##### Protein 3D structure for docking analysis

**Fig. 1.**
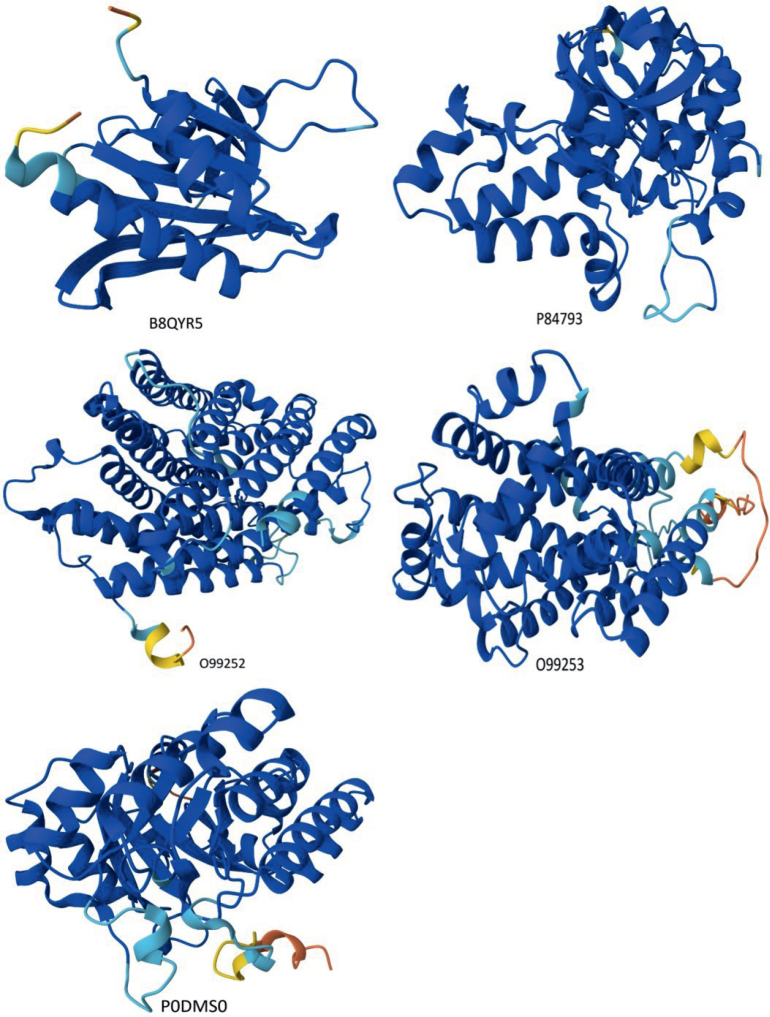
Three-dimensional structures of *Plasmodium berghei* proteins—Profilin (AFDB: B8QYR5), L-lactate dehydrogenase (AFDB: P84793), Cytochrome c oxidase subunit 1 (COI; AFDB: O99252), Cytochrome b (MT-CYB; AFDB: O99253), and Pyridoxal 5’-phosphate synthase subunit Pdx1 (AFDB: PODMsO)—retrieved from the UniProt protein database (https://www.uniprot.org/)

The three-dimensional structures of *Plasmodium berghei* target proteins—Profilin (AFDB: B8QYR5), L-lactate dehydrogenase (AFDB: P84793), Cytochrome c oxidase subunit 1 (COI; AFDB: O99252), Cytochrome b (MT-CYB; AFDB: O99253), and Pyridoxal 5’-phosphate synthase subunit (Pdx1; AFDB: P0DMS0)—were retrieved from the UniProt protein database (https://www.uniprot.org/). The 3D structures are illustrated in Figure 1.

##### Binding affinity interaction

Molecular docking was performed using the Glide Extra Precision (XP) module of Schrödinger Suite version 16.4. Ligands identified through gas chromatographymass spectrometry (GC-MS) analysis of JRLE were obtained from the PubChem BioAssay database. Ligand structures were prepared using Maestro 12.8 and LigPrep 2.4 tools. The receptor grids for each target protein were generated with a default grid size of 20 Å. Energy minimization of all ligands was conducted using the MacroModel module within the Schrödinger Suite (Schrödinger Release 2023).

##### Statistical analysis

Data were analyzed using SigmaPlot® version 11.0 (Systat Software, Inc., Chicago, IL, USA). One-way analysis of variance (ANOVA) was performed to compare differences among groups, followed by Tukey’s post hoc test for pairwise comparisons. Results are presented as mean ± standard deviation (SD), with statistical significance considered at p ≤ 0.05.

##### Ethical Approval and/or Informed Consent

Not applicable.

## Results

**Fig. 2.**
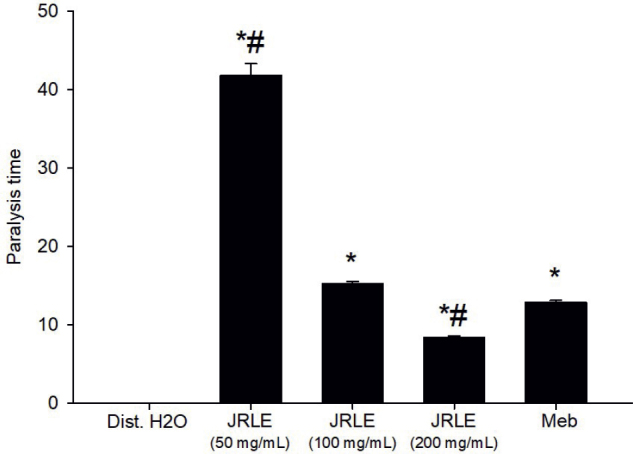
Time taken for paralysis of the earthworms, *E. fetida,* in various treatments. * Significance change with respect to those treated with dist. H_2_O, #Significance change compared to those treated with mebendazole.

**Fig. 3.**
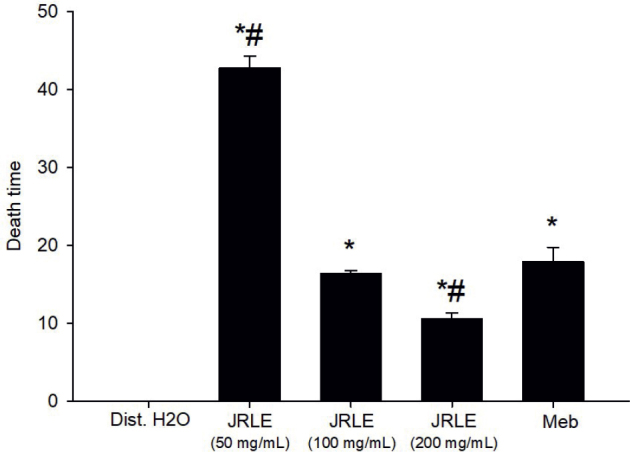
Time taken for the Death of the earthworms, *E. fetida*, in various treatments. * Significance change with respect to those treated with dist. H_2_O, #Significance change compared to those treated with mebendazole.

JRLE exhibited anthelmintic activity comparable to that of the reference drug, Mebendazole, against adult *E. fetida* worms. Paralysis and death times were recorded, with results presented in Figures 2 and 3, over a 40-minute observation period. No paralysis was observed in the control group treated with distilled water. Among the extract concentrations tested, the highest dose of JRLE (200 mg/ml) induced paralysis most rapidly (8.43 ± 0.21 min) and caused death in the shortest time (10.56 ± 0.78 min) for nearly all worms. In comparison, Mebendazole (10 mg/ml) produced paralysis and death at 12.90 ± 0.21 min and 17.93 ± 1.82 min, respectively. Lower JRLE concentrations also demonstrated significant anthelmintic effects.

**Fig. 4.**
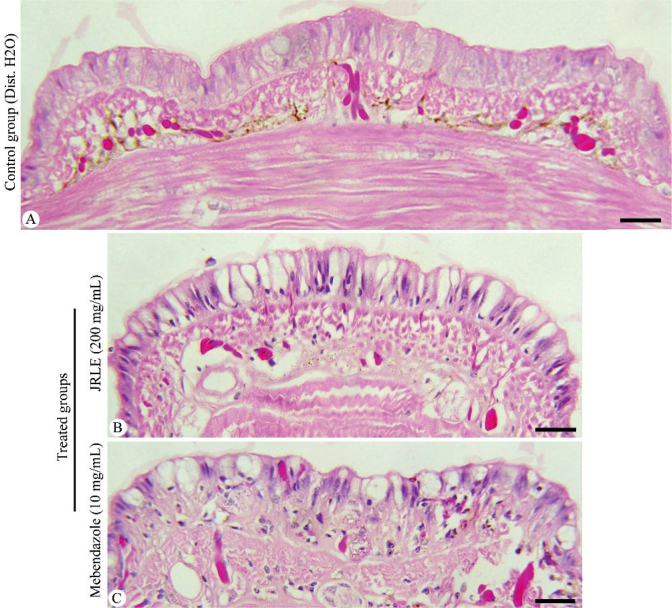
Histology of the cuticle of the earthworms, *E. fetida,* following various treatments. (A) The control group. (B) 200 mg/mL JRLE. (C) 10 mg/ml Mebendazole. Sections were stained with H&E. Scale bar = 10μm.

Microscopic observations revealed that, in the control group (distilled water), the cuticular and epidermal layers of *E. fetida* worms appeared intact and continuous, with well-defined epithelial cells and a normal muscular arrangement. No signs of tissue disruption or degeneration were observed, indicating healthy and unaltered worm morphology (Fig. 4A). In contrast, worms exposed to JRLE exhibited moderate histological alterations, including irregular epithelial structures, swelling, and increased cuticle thickness. Some cellular disorganization and vacuolation were evident, suggesting early degenerative changes induced by the extract’s bioactive components (Fig. 4B). Pronounced disruption of the cuticle and epithelial layers was observed in worms treated with Mebendazole, along with severe tissue damage and cellular lysis (Fig. 4C).

The molecular docking study of the fifteen tested compounds to different *P Berghei* proteins indicated that 3-O-Methyl-d-glucose exhibited the highest binding affinity with the protein active sites of Profilin, L-lactate dehydrogenase, and Pyridoxal 5’-phosphate synthase subunit, with docking scores of -6.44, -7.582, and -9.103 kcal/mol, respectively. Whereas, 4H-Pyran-4-one, 2,3-dihydro-3,5-dihydroxy-6-methyl-showed high binding affinity with the protein active site of the previously mentioned proteins, with docking scores equal to -4.494, -5.611, and -7.568 kcal/mol, respectively. In contrast, γ-Sitosterol and Stigmasterol showed higher binding affinity with the protein active site of Cytochrome b, with docking scores equal to -8.392 and -7.766 kcal/mol, respectively. Additionally, Heptanediamide, N, N’-di-benzoyloxy-showed high binding affinity with the protein active site of Cytochrome c oxidase subunit 1, with docking scores equal to -9.799 kcal/mol (Table 1 and Fig. 5a-i).

**Table 1.** The docking scores and hydrogen bonds between the top ligands with different proteins.

Compound name	PubChem Compound CID	Protein	Free energy of binding (Kcal/mol)	Residues involved in bonding	H-bonds distance (Å)	Number of bonds
		Profilin	-6.440	Lys^72^(2), Thr^73^, Thr^75^(2), As^p^^9^1	2.02,1.79,2.21,2.19,2.09,2.1	6H bonds
3-O-Methyl-d-glucose	8973	L-lactate dehydrogenase	-7.582	Asn^127^(2), Arg^158^, His^182^, Ser^234^, Pro^235^	1.67, 2.06, 1.79, 1.97, 1.94, 2.09 2.15, 2.01, 1.81, 1.89, 2.24,	6H bonds
		Pyridoxal 5’-phosphate synthase subunit	-9.103	Asp^27^,Lys^84^, Gly^156^, Gly^217^, lie^218^, Phe^238^, Gly^238^	1.93,2.14	7H bonds
		Profilin	-4.494	Lys^72^, Asp^91^,Gly^92^	2.44,2.12,1.9	3H bonds
4H-Pyran-4-one, 2,3-dihydro-3,5-dihydroxy-6-methyl-	119838	L-lactate dehydrogenase	-5.611	Arg^95+(S)^,His^182^,Arg^158+(S)^	2.4,1.92,2.25	3H bonds +2 (S)
		Pyridoxal 5’-phosphate synthase	-7.568	Gly^156^, Gly^217^, Ser^239^	2.08,1.82,1.94	3H bonds
γ-Sitosterol	457801	- Cytochrome b	-8.392	- Phe^284^	2.66	IHbond
St¡gmasterol	5280794		-7.766		1.84	IHbond
Heptanediamide, N,N’-dibenzoyloxy-	569848	Cytochrome c oxidase subunit 1	-9.799	Trp^131^(2), His^248^, His^382^, Arg^446^(2), Tyr^250^(P)	1.82,2.47,2.21,1.98,2.06,2.71	6H Bond +1 (P)

1 (S); salt bridges, (P); Pi-Pi stacking, the number between brackets means the residues may have many bonds

**Fig. 5a.**
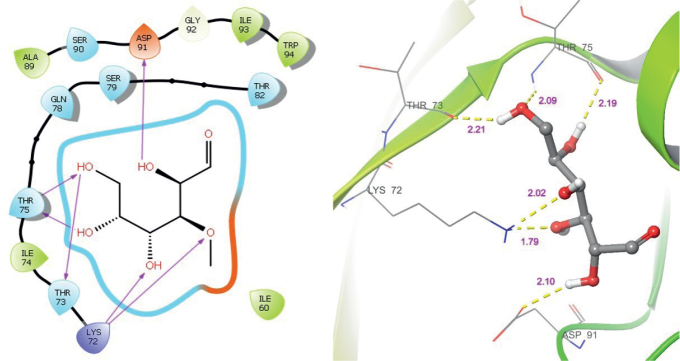
The 2D and 3D interaction between Profilin and 3-O-Methyl-d-glucose. All hydrogen bonds are represented as yellow dashes, aromatic hydrogen bonds represented as cyan blue dashes, and pi-pi stacking represented as green dashes.

**Fig. 5b.**
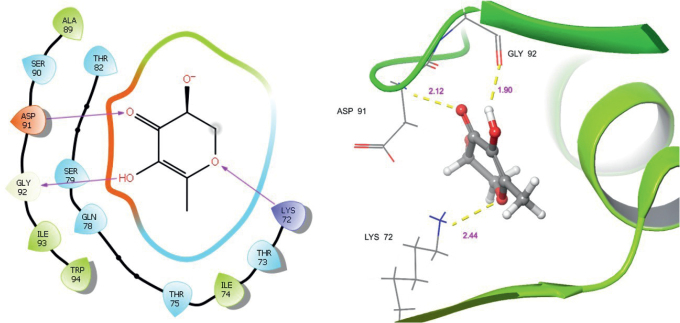
The 2D and 3D interaction between Profilin and 4H-Pyran-4-one, 2,3-dihydro-3,5-dihydroxy-6-methyk All hydrogen bonds are represented as yellow dashes, aromatic hydrogen bonds represented as cyan blue dashes, and pi-pi stacking represented as green dashes.

**Fig. 5c.**
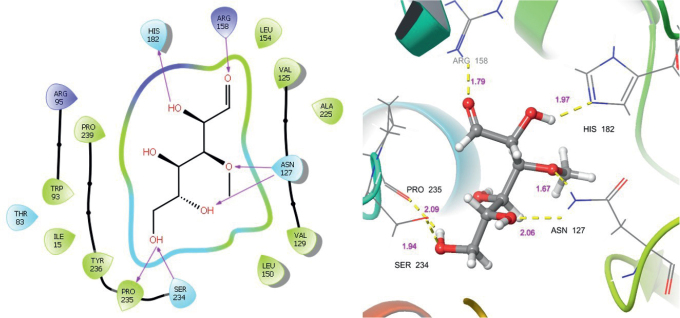
The 2D and 3D interaction between L-lactate dehydrogenase and 3-O-Methyl-d-glucose. All hydrogen bonds are represented as yellow dashes, aromatic hydrogen bonds represented as cyan blue dashes, and pi-pi stacking represented as green dashes.

**Fig. 5d.**
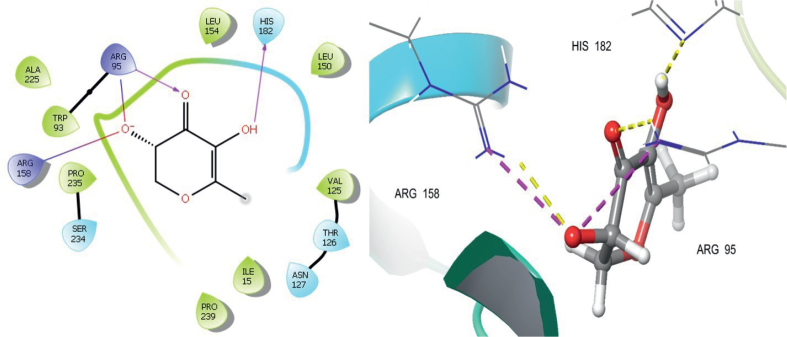
The 2D and 3D interaction between L-lactate dehydrogenase and 4H-Pyran-4-one, 2,3-dihydro-3,5-dihydroxy-6-methyl-. All hydrogen bonds are represented as yellow dashes, aromatic hydrogen bonds represented as cyan blue dashes, and pi-pi stacking represented as green dashes.

**Fig. 5e.**
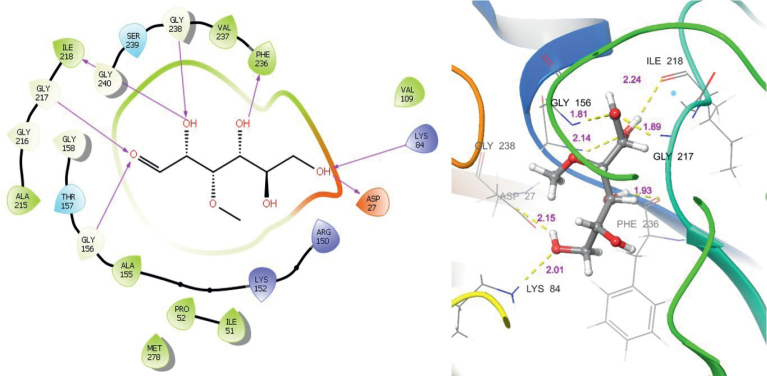
The 2D and 3D interaction between Pyridoxal 5’-phosphate synthase and 3-O-Methyl-d-glucose. All hydrogen bonds are represented as yellow dashes, aromatic hydrogen bonds represented as cyan blue dashes, and pi-pi stacking represented as green dashes.

**Fig. 5f.**
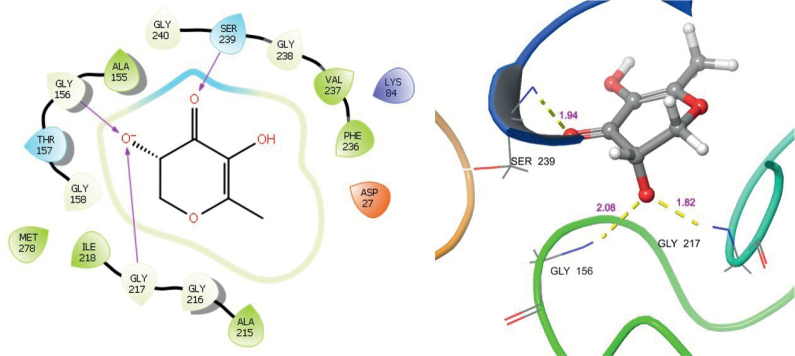
The 2D and 3D interaction between Pyridoxal 5’-phosphate synthase and 4H-Pyran-4-one, 2,3-dihydro-3,5-dihydroxy-6-methyl-. All hydrogen bonds are represented as yellow dashes, aromatic hydrogen bonds represented as cyan blue dashes, and pi-pi stacking represented as green dashes.

**Fig. 5g.**
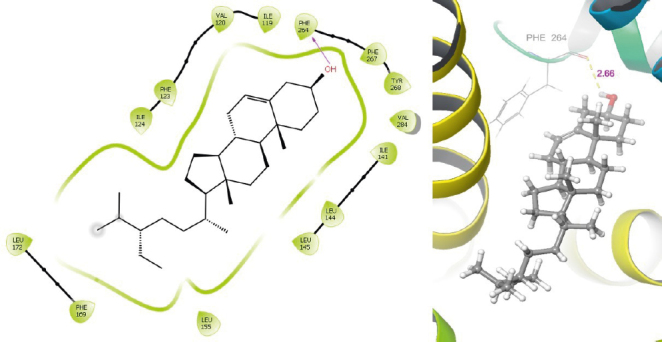
The 2D and 3D interaction between Cytochrome b synthase and γ-Sitosterol. All hydrogen bonds are represented as yellow dashes, aromatic hydrogen bonds represented as cyan blue dashes, and pi-pi stacking represented as green dashes.

**Fig. 5h.**
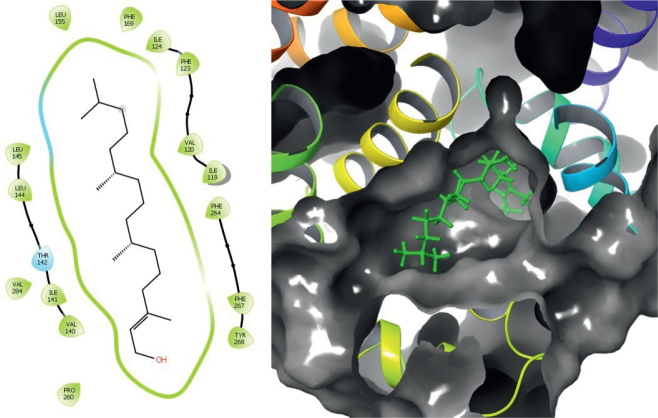
The 2D and 3D interaction between Cytochrome b synthase and Stigmasterol. There is no interaction represented in 2D as the ligand has a good confirmation inside the receptor grid, which is reflected in a high docking score. All hydrogen bonds are represented as yellow dashes, aromatic hydrogen bonds represented as cyan blue dashes, and pi-pi stacking represented as green dashes.

**Fig. 5i.**
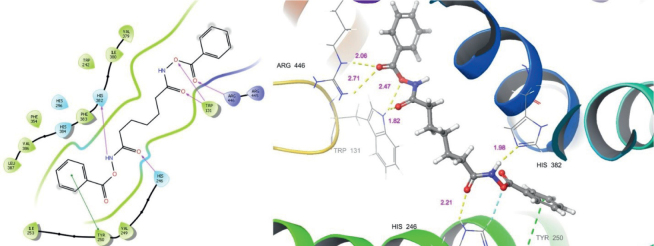
The 2D and 3D interaction between Cytochrome c oxidase subunit and Heptanediamide, N,N’-di-benzoyloxy-. All hydrogen bonds are represented as yellow dashes, aromatic hydrogen bonds represented as cyan blue dashes, and pi-pi stacking represented as green dashes.

The hydrogen (H) bonding interactions in the best docking are also described in Table 1 and Figure 5a-i. The results showed that the maximum total number of hydrogen (H) bonds between tested compounds and the Pyridoxal 5’-phosphate synthase subunit protein active site was observed with 3-O-Methyl-d-glucose, which forms 7 H-bonds, followed by interaction with Profilin and L-lactate dehydrogenase proteins active site, which forms 6 H-bonds for each. Whereas, 4 H-Pyran-4-one, 2,3-dihydro-3,5-dihydroxy-6-methylreacts with L-lactate dehydrogenase protein active site to form 3 H-bonds and 2 salt bridges, followed by Profilin and Pyridoxal 5’-phosphate synthase subunit to form 3 H-bonds for each of them. While Heptanediamide, N, N’-di-benzoyloxyinteracts with Cytochrome c oxidase subunit 1 and forms 6 H-bonds and pi-pi stacking, followed by the lower number of H-bonds between tested compounds, interaction of both Stigmasterol and γ-Sitosterol with Cytochrome b protein active site to form 1 H-bond for each.

## Discussion

Parasitic infections, particularly helminthiasis, cause significant health and economic burdens in both humans and livestock. Conventional anthelmintic therapies have shown declining effectiveness due to adverse side effects and the emergence of drug-resistant parasite strains (Chartier *et al*., 2001). These challenges have prompted increasing interest in plant-derived alternatives, which are generally safer, more accessible, and less prone to resistance development (Coles, 1997). Medicinal plants provide a rich source of bioactive compounds with antiparasitic potential (Enechi *et al*., 2019). In particular, *J. regia* leaves contain diverse phytochemicals with demonstrated antioxidant, antimicrobial, anti-inflammatory, and gastroprotective activities (Dabburu *et al*., 2012; Zakavi *et al*., 2013; Soto-Maldonado *et al*., 2019; Igbayilola *et al.,* 2022; Elouafy *et al*., 2023; Ara *et al*., 2023). Recent studies have highlighted natural compounds as promising candidates for the development of novel anthelmintic agents (Abu Hawsah *et al.,* 2023; Al-Shaebi *et al*., 2023, 2025), providing a rationale for investigating the pharmacological potential of *J. regia* leaf extracts in the present study.

Because earthworms and certain intestinal roundworms that infect humans share physiological similarities, *E. fetida* is widely used as a model organism to evaluate anthelmintic activity (Al-Shaebi *et al*., 2025). The worm’s tegument serves as a critical protective barrier, maintaining moisture balance and providing defense against external injury. In the present study, the anthelmintic efficacy of *J. regia* leaf extract (JRLE) was compared with that of the standard drug mebendazole. Microscopic analysis revealed that JRLE induced morphological alterations and structural damage in *E. fetida* similar to those caused by mebendazole. These findings align with those of Das *et al*. (2011), who observed comparable effects of walnut leaf extract on adult Indian earthworms (*Pheretima posthuma*). Quantitative microscopic assessment showed a significant reduction in cuticle thickness and body segment length in worms exposed to varying concentrations of JRLE relative to mebendazole. Such effects may be attributed to the pharmacokinetic properties of both the extract and the reference drug, which influence drug concentration at the parasite site through modulation of metabolic pathways, tissue distribution, excretion, and absorption time (Codina *et al*., 2025). Consistent results were also reported by Khalil *et al*. (2022), who demonstrated the anti-schistosomal potential of the walnut-derived compound juglone, as evidenced by reductions in worm burden and egg count. Collectively, these findings reinforce the enduring value of natural products as a foundation for novel drug discovery.

The current findings indicate that the extract exerts a rapid paralytic and lethal effect on worms, with the highest tested concentration (200 mg/ml) inducing paralysis and death at 8.43 ± 0.21 min and 10.56 ± 0.78 min, respectively, demonstrating greater potency than mebendazole. This enhanced activity may be attributed to the diverse bioactive constituents of JRLE, including phenolic acids, flavonoids, tannins, saponins, and sterols, which are known for their broad therapeutic properties. Available evidence suggests that the external surface of helminths serves as the primary site for the absorption of various chemical compounds, including anthelmintic agents. For these compounds to exert their effects, they must traverse cell membranes to reach specific receptor sites within the parasite’s biophase (Mottier *et al.,* 2006). Similarly, Kelly *et al*. (1975) reported that particle size influences the anthelmintic efficacy of mebendazole against *Nippostrongylus brasiliensis* in rats.

Molecular docking is an essential computational approach used in computer-aided drug design and structural molecular biology to predict how two molecules—such as a compound and a target enzyme—interact in three-dimensional space (Soureshjani *et al.,* 2015). This technique enables the modeling of interactions between small molecules, such as mebendazole, and the parasite target proteins (Dong *et al.,* 2025). In the present study, molecular docking analysis offered insights into how bioactive constituents of walnut extract might interact with the target proteins of *P berghei*, indicating possible therapeutic effects that warrant further experimental validation. Thus, molecular docking provides valuable insights for the discovery of novel natural antiplasmodial agents by accelerating drug development and elucidating protein-ligand interactions and structural dynamics (Suh *et al*., 2021).

In this investigation, identifying the active phytochemicals in JRLE and their strong binding affinities with *P berghei* target proteins provided significant insights into their potential therapeutic roles. Previous studies by Guleria *et al*. (2021), Owoloye *et al*. (2022), Nwonuma *et al*. (2023), and Lobato-Tapia *et al*. (2023) have emphasized that docking simulations can effectively predict binding affinities between small molecules and target proteins, facilitating antiplasmodial drug design. The advancement of computational screening methods—integrating molecular docking, molecular dynamics simulations, and various molecular property analyses— has greatly simplified the discovery of new therapeutic compounds (Evbuomwan *et al.,* 2024; Murugesan and Kaleeswaran, 2024). Collectively, the current results suggest that JRLE phytochemicals may have potential as candidate compounds for the development of safe and effective natural anthelmintic and antiplasmodial agents. Nevertheless, further *in vitro* and *in vivo* studies are essential to validate these computational predictions and to elucidate the underlying molecular mechanisms.

## Conclusion

The present study demonstrates that *J. regia* leaf extract (JRLE) exhibits significant anthelmintic activity against *E. fetida*, with the highest tested concentration (200 mg/ml) inducing rapid paralysis and death, demonstrating shorter paralysis and death times compared with the standard drug Mebendazole under the tested conditions. Histological analyses revealed that JRLE induced epithelial disorganization, swelling, vacuolation, and irregularities in the cuticle and epidermal layers, reflecting the bioactivity of its phytochemical constituents. Complementary *in silico* docking studies indicated strong binding affinities, based on docking scores, of JRLE-derived compounds with key *P. berghei* target proteins, highlighting their potential therapeutic relevance in antimalarial drug discovery. Collectively, these findings highlight the potential of JRLE as a natural anthelmintic and antiplasmodial agent, supporting the broader utility of plant-derived bioactive compounds. Further *in vitro* and *in vivo* studies are warranted to validate these outcomes and explore their translational potential.

## References

[j_helm-2026-0009_ref_001] Abu Hawsah M., Al Otaibi T., Alojayri G., Al Shaebi E.M., Dkhil M.A., Elkhadragy M.F., Al Quraishy S., Abdel Gaber R. (2023). In vitro studies for the antiparasitic activities of *Azadirachta indica* extract. Food Sci Technol.

[j_helm-2026-0009_ref_002] Agrawal J., Murthy P., Philip M., Mehrotra S., Thennarasu K., John J.P. (2010). Socio demographic correlates of subjective well being in urban India. Soc Indic Res.

[j_helm-2026-0009_ref_003] Agu P.C., Afiukwa C.A., Orji O.U., Ezeh E.M., Ofoke I.H., Ogbu C.O., Ugwuja E.I., Aja P.M. (2023). Molecular docking as a tool for the discovery of molecular targets of nutraceuticals in disease management. Sci Rep.

[j_helm-2026-0009_ref_004] Alhaiqi N.S., Afifi S.M., Mahyoub J.A., Abdel Gaber R.A., Delic D., Dkhil M.A. (2024). Anthelmintic activity of *Carica papaya* leaf extracts: insights from *in vitro* and *in silico* investigations. Comb Chem High Throughput Screen.

[j_helm-2026-0009_ref_005] Al Shaebi E.M., Abdel Gaber R., Al Hoshani N., Al Quraishy S. (2025). Evaluation of the cytotoxicity and anthelmintic activity of *Olea europaea* (stem and leaves) methanolic extract: *in vitro* investigation. Helminthologia.

[j_helm-2026-0009_ref_006] Al Shaebi E.M., Al Quraishy S., Abdel Gaber R., Maodaa S.N., Alatawi A., Alawwad S.A. (2023). Efficacy of *Teucrium polium* leaves extract as anticoccidial and anthelmintic: *in vitro* study. Arq Bras Med Vet Zootec.

[j_helm-2026-0009_ref_007] Ameen S.A., Azeez O.M., Baba Y.A., Raji L.O., Basiru A., Biobaku K.T. (2018). Anthelmintic potency of *Carica papaya* seeds against gastrointestinal helminths in Red Sokoto goats. Ceylon J Sci.

[j_helm-2026-0009_ref_008] Ara T., Shafi S., Ghazwani M., Mir J.I., Shah A.H., Qadri R.A., Hakami A.R., Khalid M., Hani U., Wahab S. (2023). In vitro potent anticancer, antifungal, and antioxidant efficacy of walnut (*Juglans regia* L.) genotypes. Agronomy.

[j_helm-2026-0009_ref_009] Challapa Mammani M.R., Tomás Alvarado E., Espinoza Baigorria A., León Figueroa D.A., Sah R., Rodriguez Morales A.J. (2023). Molecular docking and molecular dynamics simulations in relation to *Leishmania donovani*: an update and literature review. Trop Med Infect Dis.

[j_helm-2026-0009_ref_010] Chartier C., Soubirac F., Pors I., Silvestre A., Hubert J., Couquet C., Cabaret J. (2001). Prevalence of anthelmintic resistance in gastrointestinal nematodes of dairy goats under extensive management conditions in southwestern France. J Helminthol.

[j_helm-2026-0009_ref_011] Codina A.V., Indelman P., Hinrichsen L.I., Lamas M.C. (2025). Significant improvement in bioavailability and therapeutic efficacy of Mebendazole oral nano-systems assessed in a murine model with extreme phenotypes of susceptibility to *Trichinella spiralis*. Pharmaceutics.

[j_helm-2026-0009_ref_012] Coles G.C. (1997). Nematode control practices and anthelmintic resistance on British sheep farms. Vet Rec.

[j_helm-2026-0009_ref_013] Dabburu K., Kondaveeti S.B., Babu S.K. (2012). Evaluation of gastro-protective effect of the hydro-alcoholic extract of *Juglans regia* L. leaves in experimental animals. J Appl Pharm Sci.

[j_helm-2026-0009_ref_014] Das R., Mehta D.K., Gupta A. (2011). *In vitro* anthelmintic activity of leaves of *Juglans regia* against *Pheretima posthuma*. Sci Rev Chem Commun.

[j_helm-2026-0009_ref_015] Dong J., Xia L., Liu Y., Yang Q., Xu N., Ai X., Zhou S. (2025). Discovery of potential anthelmintic agents against *Gyrodactylus kobayashii* through computer-aided drug design and *in vivo* evaluation. J Fish Dis.

[j_helm-2026-0009_ref_016] Drury R.A., Wallington E.A. (1973). Carleton’s histological technique.

[j_helm-2026-0009_ref_017] Elouafy Y., El Yadini A., Mortada S., Hnini M., Harhar H., Khalid A. (2023). Antioxidant, antimicrobial, and α-glucosidase inhibitory activities of saponin extracts from walnut (*Juglans regia* L.) leaves. Asian Pac J Trop Biomed.

[j_helm-2026-0009_ref_018] Enechi O.C., Amah C.C., Okagu I.U., Ononiwu C.P., Azidiegwu V.C., Ugwuoke E.O. (2019). Methanol extracts of *Fagara zanthoxyloides* leaves possess antimalarial effects and normalize haematological and biochemical status of *Plasmodium berghei*-passaged mice. Pharm Biol.

[j_helm-2026-0009_ref_019] Evbuomwan I.O., Alejolowo O.O., Elebiyo T.C., Nwonuma C.O., Ojo O.A., Edosomwan E.U., Chikwendu J.I., Elosiuba N.V., Akulue J.C., Dogunro F.A., Rotimi D.E., Osemwegie O.O., Ojo A.B., Ademowo O.G., Adeyemi O.S., Oluba O.M. (2023). In silico modeling revealed phytomolecules derived from *Cymbopogon citratus* (DC.) leaf extract as promising candidates for malaria therapy. J Biomol Struct Dyn.

[j_helm-2026-0009_ref_020] García Bustos J.F., Sleebs B.E., Gasser R.B. (2019). An appraisal of natural products active against parasitic nematodes of animals. Parasit Vectors.

[j_helm-2026-0009_ref_021] Guleria V., Pal T., Sharma B., Chauhan S., Jaiswal V. (2021). Pharmacokinetic and molecular docking studies to design antimalarial compounds targeting Actin I. Int J Health Sci (Qassim).

[j_helm-2026-0009_ref_022] Halton E. (2004). The living gesture and the signifying moment. Symb Interact.

[j_helm-2026-0009_ref_023] Igbayilola Y.D., Aina O.S., Ogunkoya O.O., Williams O.D., Olaoye F.A. (2022). Oxidative, hepatoprotective, and anti-inflammatory responses to perinatal walnut (*Juglans regia* L.) supplemented diet in offspring of Sprague-Dawley rats. Int J Biochem Physiol.

[j_helm-2026-0009_ref_024] Kelly J., Chevis R., Goodman H. (1975). Effect of particle size on the anthelmintic efficacy of Mebendazole against *Nippostrongylus brasiliensis* in the rat. Int J Parasitol.

[j_helm-2026-0009_ref_025] Khalil R.G., Ibrahim A.M., Bakery H.H. (2022). Juglone: a novel immunomodulatory, antifibrotic, and schistosomicidal agent to ameliorate liver damage in murine *schistosomiasis mansoni*. Int Immunopharmacol.

[j_helm-2026-0009_ref_026] Lobato Tapia C.A., Moreno Hernández Y., Olivo Vidal Z.E. (2023). In silico studies of four compounds of *Cecropia obtusifolia* against malaria parasite. Molecules.

[j_helm-2026-0009_ref_027] Mottier L., Alvarez L., Ceballos L., Lanusse C. (2006). Drug transport mechanisms in helminth parasites: passive diffusion of benzimidazole anthelmintics. Exp Parasitol.

[j_helm-2026-0009_ref_028] Murugesan R., Kaleeswaran B. (2024). *In silico* drug discovery: unveiling potential targets in *Plasmodium falciparum*. Asp Mol Med.

[j_helm-2026-0009_ref_029] Ndjonka D., Rapado L.N., Silber A.M., Liebau E., Wrenger C. (2023). Natural products as a source for treating neglected parasitic diseases. Int J Mol Sci.

[j_helm-2026-0009_ref_030] Nwonuma C.O., Balogun E.A., Gyebi G.A. (2023). Evaluation of antimalarial activity of ethanolic extract of *Annona muricata* L.: an *in vivo* and *in silico* approach. J Evid Based Integr Med.

[j_helm-2026-0009_ref_031] Owoloye A.J., Ligali F.C., Enejoh O.A., Musa A.Z., Aina O., Idowu E.T., Oyebola K.M. (2022). Molecular docking, simulation, and binding free energy analysis of small molecules as PfHT1 inhibitors. PLoS One.

[j_helm-2026-0009_ref_032] Parida S., Patro V.J., Mishra U.S., Mohapatra L., Sannigrahi S. (2010). Anthelmintic potential of crude extracts and various fractions of different parts of *Pterospermum acerifolium* Linn. Int J Pharm Sci Rev Res.

[j_helm-2026-0009_ref_033] Paul A., Das T., Chowdhury M.H.U., Majumder M., Khan M.M., Emran T.B. (2024). Anthelmintic activity of pineapple: *in silico* molecular docking and molecular dynamics simulation. Res Sq.

[j_helm-2026-0009_ref_034] Schrodinger Release (2023). Protein Preparation Wizard; Epik, Maestro, SiteMap, MacroModel, Glide, LigPrep.

[j_helm-2026-0009_ref_035] Shi W., Xu N., Wang X., Vallée I., Liu M., Liu X. (2022). Helminth therapy for immune-mediated inflammatory diseases: current and future perspectives. J Inflamm Res.

[j_helm-2026-0009_ref_036] Soto Maldonado C., Vergara Castro M., Jara Quezada J., Caballero Valdés E., Müller Pavez A., Zúñiga Hasen M.E. (2019). Polyphenolic extracts of walnut (*Juglans regia*) green husk containing juglone inhibit the growth of HL-60 cells and induce apoptosis. Electron J Biotechnol.

[j_helm-2026-0009_ref_037] Soureshjani E.H., Babaheydari A.K., Saberi E. (2015). DNA methyltransferases directed anti-cancerous plant medicine (Xanthomicrol and Galloyl) based molecular docking and dynamics simulation. Comput Mol Biosci.

[j_helm-2026-0009_ref_038] Suh D., Lee J.W., Choi S., Lee Y. (2021). Recent applications of deep learning methods on evolution-and contact-based protein structure prediction. Int J Mol Sci.

[j_helm-2026-0009_ref_039] Zakavi F., Hagh L.G., Daraeighadikolaei A., Sheikh A.F., Daraeighadikolaei A., Shooshtari Z.L. (2013). Antibacterial effect of *Juglans regia* bark against oral pathologic bacteria. Int J Dent.

